# Outcome of Treatment of Anterior Vaginal Wall Prolapse and Stress Urinary Incontinence with Transobturator Tension-Free Vaginal Mesh (Prolift) and Concomitant Tension-Free Vaginal Tape-Obturator

**DOI:** 10.1155/2009/341268

**Published:** 2008-12-25

**Authors:** Ashraf Abou-Elela, Essam Salah, Haitham Torky, Sameh Azazy

**Affiliations:** ^1^Department of Urology, Cairo University, Cairo, Egypt; ^2^Department of Gynaecology, October 6 University, October 6 City, Egypt; ^3^Department of Gynaecology, Well-Care Medical Centre, Abudhabi, United Arab Emirates

## Abstract

*Objective*. It is to assess the feasibility, effectiveness, and safety of transobturator tension-free vaginal mesh (Prolift) and concomitant tension-free vaginal tape-obturator (TVT-O) system as a treatment of female anterior vaginal wall prolapse associated with stress urinary incontinence (SUI). *Patients and Methods*. Between December 2006 and July 2007, 20 patients with anterior genital prolapse and voiding dysfunction were treated with the transobturator tension-free vaginal mesh (Prolift) and concomitant tension-free vaginal tape-obturator (TVT-O). Sixteen patients had stress urinary incontinence and 4 patients were considered at risk for development of de novo stress incontinence after the prolapse is repaired. All patients underwent a complete urodynamic assessment. All the patients underwent pelvic examination 4–6 weeks after the operation, and anatomical and functional outcomes were recorded. *Results*. Twenty cystocoeles were repaired: 6 grade II, 12 grade III, and 2 grade IV. There were no vessel or bladder injuries. Eighteen patients had optimal anatomic results and 2 patients had persistent asymptomatic stage I prolapse. *Conclusion*. These preliminary results suggest that Prolift system offers a safe and effective treatment for female anterior vaginal wall prolapse. However, a long-term followup is necessary in order to support the good result maintenance.

## 1. Introduction

Genital prolapse affects the quality of life of women [1]. More than 50% of multiparas suffer this problem [2]. The estimated lifetime risk of surgery for prolapse or incontinence is 11%, with one of three patients requiring more than one surgical repair [3]. Pathogenesis of genital prolapse is the result of the weakness of any or all of the pelvic support structures, that is, levator ani muscle, connective tissue, uterosacral and cardinal ligaments, and rectovaginal fascia [4]. Anterior vaginal wall prolapse may coexist with disorders of micturition. Mild anterior vaginal wall prolapse usually presents few problems. As prolapse progresses, symptoms may develop and worsen, and treatment becomes indicated. The classic surgical techniques have a high recurrence rate that could be between 5 and 40% for cystocel [5, 6]. Considering pelvic organ prolapse as a hernia through the genital hiatus, nonabsorbable mesh has been advocated for high-grade genital prolapse, based on its use in general surgery for hernia repair [7]. The *perfect* mesh must be biocompatible, inert, sterile, not carcinogenous, not allergenic, and resistant. Currently, polypropylene macrospore monofilament gynecological mesh has been used as fascial strengthening, with tension-free technique, reducing the possibility of relapse. A new mesh system for treatment of genital prolapse is the system Prolift [8]. It is a wide mesh with an anchor system that provides a complete support and is applied with a minimal invasive technique. We used the new system Prolift with the objective of reviewing the safety and effectiveness for the treatment of female anterior vaginal wall prolapse.

## 2. Patients and Methods

Between December 2006 and July 2007, twenty patients affected with anterior prolapse were included in this prospective survey. All the patients were referred to our Urology Unit because of their voiding problems. Mean age was 52 years (36–76). Mean parity, 3 vaginal childbirths. Sixteen patients with anterior vaginal wall prolapse reported socially annoying type II or III urinary stress incontinence, 4 patients reported voiding difficulty and related a history of urinary incontinence that has resolved with worsening of their prolapse. These patients were considered at risk for development of de novo stress incontinence after the prolapse is repaired. Twelve patients reported symptoms related to prolapse including the sensation of a vaginal mass or bulge, pelvic pressure, low back pain, and sexual difficulty. Twelve patients were sexually active, 8 had sexual difficulty (Table 1). The examination was first performed with the patient supine in lithotomy position. A retractor or Sims speculum was used to depress the posterior vagina to aid in visualizing the anterior vagina. After the resting examination, the patient was instructed to strain down forcefully or to cough vigorously. During this maneuver, the order of descent of the pelvic organs and their relationship at the peak of straining were noted. If physical findings did not correspond to symptoms or if the maximum extent of the prolapse could not be confirmed, the woman was reexamined in the standing position.

A urinalysis was performed to evaluate for urinary tract infection. In all patients, whether with stress urine incontinence or voiding dysfunction and probability to develop incontinence after the repair, a complete urodynamic assessment was performed. In women with grade III and IV prolapse, the urethral function was checked after the prolapse is repositioned. Urodynamic evaluation confirmed the diagnosis of SUI in 16 patients, and in the remaining 4 patients after repositioning of the prolapse Valsalva, leak point pressure was obtained with the patient standing, filled to bladder capacity, and the urethral catheter removed. It was defined as the lowest abdominal pressure occurring during the Valsalva maneuver that resulted in leakage of fluid per urethra. Based on preoperative urodynamics, we defined genuine stress incontinence as a Valsalva leak point pressure of greater than 50 cm H_2_O.

Completeness of bladder emptying was measured with a timed void followed by bladder ultrasonography to measure residual urine volume. In menopauses women with atrophic status, the vaginal mucosa was prepared using local estriol 21 days before the procedure.

**Surgical Technique**

All patients received spinal anesthesia and cephalosporin and metronidazol as antibiotic prophylaxis. The patient is placed in the lithotomy position and her thighs flexed approximately 90 degrees. A 16 Fr Foley catheter is placed to empty the bladder.

**Figure 1:**
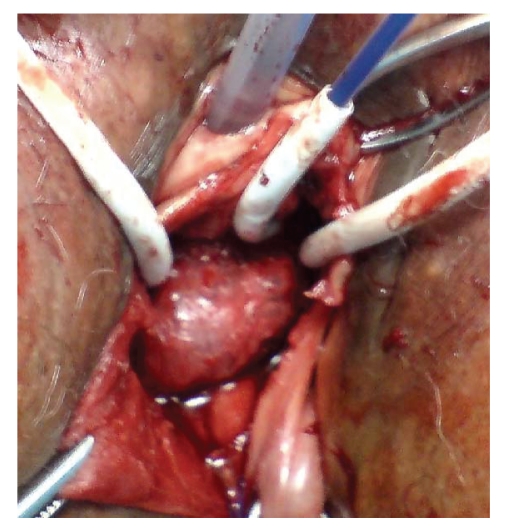
Bladder dissected from the anterior vaginal wall and 3 of the 4 cannulas and retrieval device in Prolift anterior mesh.

**Figure 2:**
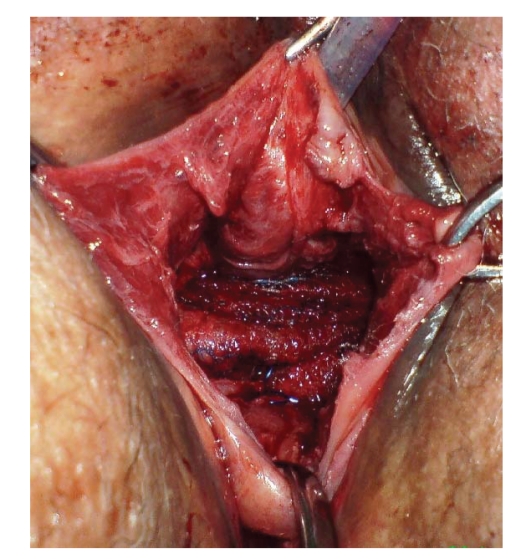
Prolift in place after reduction of the cystocoele.

The technique involves implantation of a large sheet of high-porosity monofilament polypropylene “tension-free” mesh featuring anterior intervesicovaginal prosthesis. The anterior prosthesis is retained by two nonsecured bilateral transobturator arms anteriorly at a point 1 to 2 cm from the proximal arcus tendineus fasciae pelvis and posteriorly at a point 1 to 2 cm distal from the arcus tendineus fasciae pelvis (Figure 1). Four cannulas are inserted at the fixation points with the use of a single trocar needle; the mesh arms are retrieved with a plastic loop and secured after implantation of the mesh and removal of the cannulas (Figure 2). In all patients, a tension-free vaginal tape-obturator (TVT-O) procedure was performed in all cases through a separate incision at the mid urethra after the mesh procedure for proper positioning and to avoid displacement. The hospital discharge was at 48 hours. All intraoperative and postoperative complications were recorded. All the patients underwent pelvic examination 4–6 weeks after the operation, and anatomical and functional outcomes were recorded. The patients estimated the severity of their prolapse symptoms before and after the operation, together with lifestyle and urinary and sexual discomfort, on a visual analog scale with a score range of 0 to 10 (0 corresponding to no discomfort, and 10 to maximum discomfort). Postoperative followup consisted of clinical control at 6 weeks and 6 months assessing the results of the prolapse treatment. Also, a satisfaction questionnaire was required.

## 3. Results

Twenty cystocoeles were repaired: 6 grade II, 12 grade III, and 2 grade IV. In all patients, we carried out simultaneous procedure TVT-O for type II and III stress incontinence.

Surgical mean time for anterior Prolift and TVT-O was 45 minutes (30–55 minutes). Anterior Prolift was 40 minutes (range 30–50). No intraoperative complications were registered. There were no vessel or bladder injuries. During the immediate postoperative period, a case of moderate perivesical hematoma was registered. The patient was a 62-year-old woman operated with anterior Prolift due to a grade II cystocoele and TVT-O. Oral analgesic and anti-inflammatory treatment was instituted with complete resolution. No reoperation was required.

Regarding to the satisfaction grade at 6 weeks and 6 months, all the patients answered are to be satisfied. The lifestyle discomfort score and the urinary discomfort score fell significantly after surgery while the sexual discomfort score did not change significantly after the operation in sexually active patients. One patient experienced dyspareunia after the procedure.

**Figure 3:**
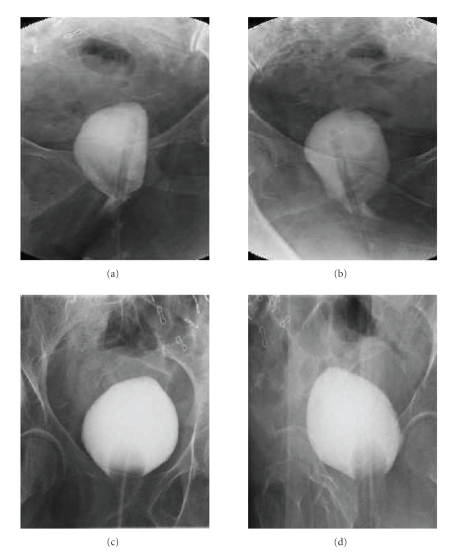
(a), (b) Preoperative ascending cystogram of a patient with G III cystocoele and severe SUI. (c), (d) Postoperative ascending cystogram of the same patient, 6 weeks after Prolift and TVT-O insertion.

Eighteen patients had optimal anatomic results (Figure 3) and 2 patients had persistent asymptomatic stage I prolapse. Mean following time was 8 months (6–14 months). No infection or rejection of the mesh occurred during followup. With regard to the simultaneous correction of the urine incontinence, in all the patients, it was successful. A patient was considered cured of SUI if she reported no leakage and satisfaction with the surgical outcome on questionnaire analysis, plus no urine loss on provocative physical examination. No cases of urine incontinence were observed after the procedure.

## 4. Discussion

Anterior vaginal wall prolapse occurs commonly and may coexist with disorders of micturition. Mild anterior vaginal wall prolapse often occurs in parous women but usually presents few problems. As prolapse progresses, symptoms may develop and worsen, and treatment becomes indicated.

Nichols and Randall [9] described two types of anterior vaginal wall prolapse: distention and displacement. Distention was thought to result from overstretching and attenuation of the anterior vaginal wall, caused by overdistention of the vagina associated with vaginal delivery or atrophic changes associated with aging and menopause. The distinguishing physical feature of this type was described as diminished or absent rugal folds. The other type, displacement, was attributed to pathologic detachment or elongation of the anterolateral vaginal supports to the arcus tendineus fasciae pelvis, resulting in descent of the anterior segment with the rugae intact.

Another theory ascribes most cases of anterior vaginal wall prolapse to disruption or detachment of the lateral connective tissue attachments at the arcus tendineus fasciae pelvis, resulting in a paravaginal defect and corresponding to the displacement type discussed above. This was described by Richardson et al. in 1976 [10]. These researchers described transverse defects, midline defects, and defects involving isolated loss of integrity of pubourethral ligaments. Transverse defects were said to occur when the “pubocervical” fascia separated from its insertion around the cervix, whereas midline defects represented an anteroposterior separation of the fascia between the bladder and vagina.

More recent innovative approaches for anterior vaginal wall repair anchor an allograft, xenograft, or polypropylene mesh without tension via strips placed through the obturator foramen with a special device (Anterior Prolift, Gynecare). These techniques await safety and efficacy studies but are increasing in use. The advantage of this approach is that all defects (central, lateral, proximal, and distal) can be treated in a time-efficient manner.

Placement of nonabsorbable mesh into an anterior vaginal wall prolapse repair is a promising but more controversial variation. Polypropylene mesh has limited foreign body reaction in general and is probably the best choice. Technique variations include mesh overlays, modified four-corner attachments, transobturator attachments, and anterior flaps as part of an apical mesh procedure. Cure rates appear high but comparative trials with more traditional sutured repairs have not been done. The goal of the procedure is to reestablish level II support of the vagina. In our preliminary study of patients with stage II, III, or IV prolapse, 90% of women had an optimal anatomic outcome, while the remaining 10% had persistent but asymptomatic anterior vaginal wall prolapse. These results are in keeping with those of Migliari, who reported the persistence of asymptomatic grade 1 cystocele after tension-free vaginal mesh repair of anterior vaginal wall prolapse (grade 3 in 25% of cases) [11].

In our study with a relatively short-term followup, the subjective anterior vaginal wall prolapse cure rate was 100%. A significant improvement in quality of life was also obtained. Ruparelia et al. reported a patient satisfaction rate of 85% twenty months after anterior vaginal wall repair with porcine skin collagen implants [12].

Short-term patient satisfaction rates range from 74% to 100% after vaginal wall repair with nonabsorbable synthetic mesh [6, 13, 14]. Functional results should be considered as important as anatomic outcomes. All urinary symptoms decreased after surgery. The risk of new functional symptoms was low, in particular, no de novo dyspareunia was observed. There are conflicting reports regarding sexual function in patients who underwent transvaginal polypropylene mesh surgery for pelvic organ prolapse [7, 15]. The surgical technique may be one of many underlying causes. In fact, wide proximal vaginal dissection or dissection of prolapsed vagina after hysterectomy could cause injury to distal pelvic perineal and cavernous nerves, which reach the clitoral tissue, and lead to difficulty in achieving female orgasm or satisfactory sexual activity. However, we believe that by avoiding plication of fascial tissue or levator muscles and also by avoiding excision of vaginal skin or mucosa, excessive folding and scarring in the anterior and posterior compartments can be prevented.

Salomon et al. used the transobturator route to secure the anterior end of the implant for the repair of genital prolapse [16, 17], and reported that among the nine patients who complained of cystocele with SUI, seven were cured with no further treatment [16]. David-Montefiore et al. found that of the 13 patients with preoperative SUI associated to genital prolapse, 8 were cured following surgery [17]. The remaining five women had improvement and did not require additional surgery [17]. In our study, we found that transvaginal mesh with concomitant anti-incontinence TVT-O procedure significantly decreased SUI and possibility of incontinence after the correction of prolapse. All 16 patients with preoperative SUI were cured and no patient of the remaining 4 developed incontinence after correction. These results are in accordance those of Solà Dalenz et al. who demonstrated the possibility to treat the prolapse and the incontinence simultaneously with two meshes without adding morbidity [18]. We believe that the strategy for management of concomitant SUI improves the results by the restoration of the defective connection between the urethra and the vagina and, therefore, via the reinforcement of the suburethral hammock.

High recurrence rates of anterior vaginal wall prolapse with or without the use of absorbable mesh have prompted most surgeons to use non-absorbable materials such as polypropylene. However, polypropylene can cause foreign-body reactions, infection, and erosion through the vagina. Reported rates of erosion associated with polypropylene range from 2.1 to 25% [6, 19, 20]. A significant number of these patients require reoperation for mesh removal. Analysis of the first 100 Prolift vaginal mesh procedures revealed a 17.5% erosion rate, which fells to 2.7% with limitation of the number and extent of colpotomies and avoidance of concomitant hysterectomy and perineal incisions [20]. In our study, creation of vaginal flaps that are thicker with an attached fibromuscularis, antibiotic prophylaxis, and vaginal preparation with estriol reduced the mesh erosion rate.

Sexual function may be positively or negatively affected by vaginal operations for anterior vaginal wall prolapse. The current popularity of synthetic or allograft mesh to augment vaginal prolapse repairs could improve sexual function if cure rates improve or could worsen function if vaginal stiffness, mesh erosions, or draining sinuses result. More data with careful followup after surgery are needed.

Efficacy and safety long-term trials are paramount prior to widespread adoption. Emerging techniques must be compared with gold-standard procedures in well-designed, long-term trials for anatomic and functional outcomes.

## 5. Conclusions

These preliminary results suggest that tension-free vaginal mesh (Prolift) placement by the transobturator route is a safe and effective treatment for symptomatic anterior vaginal wall prolapse. Stress urinary incontinence may be treated and avoided by simultaneously placing a transobturator TVT-O without adding morbidity However, further studies are warranted to determine long-term outcomes and to compare this approach with previously accepted surgical procedures.

## Figures and Tables

**Table 1:** Demographic and clinical characteristics of the patients.

Characteristics	Patients
Median age (y) (range)	52 (36–76)
Median parity (range)	3 (0–9)
Median BMI (range)	31.2 (21.4–41.5)
Prior surgery for prolapse including hysterectomy (*n*, %)	3 (15)
Prior hysterectomy for benign tumor (*n*, %)	4 (20)
Stage of prolapse	
* * * *Stage II (*n*, %)	6 (30)
* * * *Stage III (*n*, %)	12 (60)
* * * *Stage IV (*n*, %)	2 (10)
Urinary incontinence (*n*, %)	16 (80)
